# Cytochrome P450 26A1 Modulates the Polarization of Uterine Macrophages During the Peri-Implantation Period

**DOI:** 10.3389/fimmu.2021.763067

**Published:** 2021-10-12

**Authors:** Wen-Heng Ji, Dan-Dan Li, Dan-Ping Wei, Ai-Qin Gu, Ying Yang, Jing-Pian Peng

**Affiliations:** ^1^State Key Laboratory of Stem Cell and Reproductive Biology, Institute of Zoology, Chinese Academy of Sciences, Beijing, China; ^2^University of Chinese Academy of Sciences, Beijing, China

**Keywords:** peri-implantation period, embryo implantation, CYP26A1, uterine macrophages polarization (M1/M2), inflammatory response

## Abstract

Uterine M1/M2 macrophages activation states undergo dynamic changes throughout pregnancy, and inappropriate macrophages polarization can cause adverse pregnancy outcomes, especially during the peri-implantation period. Our previous studies have confirmed that Cytochrome P450 26A1 (CYP26A1) can affect embryo implantation by regulating uterine NK cells and DCs. The aim of this study was to investigate whether CYP26A1 regulates the polarization of uterine macrophages in early pregnancy. Here, we observed that *Cyp26a1* was significantly upregulated in M1 as compared with M2 of uterine macrophages, Raw264.7 and iBMDM. Knockdown of CYP26A1 in mice uterine significantly decreased the number of embryo implantation sites and the proportion of CD45^+^F4/80^+^CD206**^−^** M1-like uterine macrophages. Primary uterine macrophages treated with anti-CYP26A1 antibody expressed significantly lower levels of M1 markers *Nos2*, *Il1b*, *Il6* and *Tnf-a*. In CYP26A1 knockout Raw264.7 cells, the protein levels of M1 markers TNF-α, IL-6 and CD86 were significantly decreased as compared with the wild type cells. Moreover, CYP26A1 deficiency decreased the ability to produce nitric oxide and increased the phagocytosis capacity of Raw264.7 cells under M1 stimulation state. The re-introduction of CYP26A1 partially reversed the polarization levels of M1 in CYP26A1 knockout Raw264.7 cells. CYP26A1 may regulate the polarization of uterine macrophages to M1 through *Stap1* and *Slc7a2*. In summary, these results indicate that CYP26A1 plays a significant role in macrophage polarization, and knockdown of CYP26A1 can cause insufficient M1 polarization during the peri-implantation period, which has adverse effects on blastocyst implantation.

## Introduction

Pregnancy is a very important biological event. With in-depth understanding of the immunology of pregnancy, Medawar’s hypothesis about maternal immune tolerance to semi-alogenous fetuses sixty years ago was incorrect or insufficient (1). Actually, the maternal immune system plays an important regulatory function in the entire gestation process (2). During the first-trimester pregnant decidua, 30~40% of the cells are leukocytes, among which NK cells (50~70%), macrophages (~20%) and T cells (10~20%) are primarily subsets of leukocytes, whereas DCs, B cells and NKT are rare (3–5). As the second largest leukocyte population in the human non-pregnant endometrium and decidua, macrophages are present in all stages of pregnancy and play a vital role in the establishment and maintenance of normal pregnancy (6). They participate in the blastocyst implantation and trophoblast invasion (4, 7–9), remodeling of spiral arteries (10, 11), clearance of apoptotic cells and pathogenic microorganisms (12–14), delivery (15), and post-partum uterine recovery (16, 17).

As the most plastic immune cells, macrophages can change their phenotypes rapidly and acquire specialized functions according to the diverse microenvironment signals (18). According to different inducers and their involvement in Th1 and Th2 immune responses, macrophages can be divided into two categories: M1 (classically activated macrophages) and M2 (alternatively activated macrophages), which are two terminals of a functional state spectrum (19, 20). M1 macrophages secrete high levels of pro-inflammatory cytokines and mediators, which have a central role in antimicrobial killing and inflammatory response; while M2 macrophages are involved in many important biological processes such as inflammation resolution, wound healing and tissue remodeling (21). The activation state of uterine M1/M2 macrophages is precisely regulated and presents dynamic changes at different stages of pregnancy (21, 22). Disruption of the balance of uterine M1/M2 macrophages will cause a series of adverse pregnancy outcomes such as preeclampsia and intrauterine infections (23, 24). Although the dynamic balance of uterine M1/M2 macrophages is important for maintaining maternal-fetal immune homeostasis and successful pregnancy, little is known about the key molecules that regulate the polarization of macrophages during pregnancy.

CYP26A1 is a member of cytochrome P450 protein family, which participates in many biological processes, especially in embryonic development (25–27). CYP26A1 knockout mice largely die during mid-late gestation, accompanied by some major morphogenetic defects (25, 26). In our previous study, we have found that CYP26A1 is a differentially expressed gene in rats during the peri-implantation period by suppression subtractive hybridization (28). Further research has showed that both CYP26A1 mRNA and protein have a specific temporal and spatial expression pattern in mice uterine during blastocyst implantation period, and knockdown of CYP26A1 *in vivo* can significantly reduce the number of embryo implantation sites (29). In addition, we also have confirmed that CYP26A1 can affect embryo implantation by regulating uterine NK cells and DCs (30, 31).

Based on our previous findings, we hypothesized that CYP26A1 can affect embryo implantation by regulating the polarization of uterine macrophages. In this study, we demonstrated that knocking down CYP26A1 can disrupt the balance of uterine M1-like and M2-like macrophages, which has adverse effects on blastocyst implantation. *In vitro* studies further confirmed that CYP26A1 can regulate the polarization of macrophages towards M1 and affect the function of macrophages. Collectively, this work revealed a novel role of CYP26A1 in regulating the polarization of macrophages during the peri-implantation period.

## Materials and Methods

### Mice

BALB/c mice (8–10 weeks of age) were obtained from BeijingVital River laboratory animal center (Beijing, China) and maintained in the animal facilities at the Institute of Zoology, Chinese Academy of Sciences (Beijing, China). All experiments were conducted in accordance with the ethical guidelines of the Animal Care and Use Committee of Institute of Zoology, Chinese Academy of Sciences. Female and male mice lived together at a 1:1 ratio in a cage overnight, and the appearance of vaginal plug on the next morning was identified as gestational day 1 (GD1).

### Morpholino Antisense Oligonucleotides (MOs) Knockdown Mice

MOs were administered by intrauterine injection as previously described with some minor adjustments (29, 32). Cyp26a1 MO (5’-CATGGCACGCTTCAGCCTCCCGCGC-3’) and Random Control MO (5’-25-N-3’) used in this study were synthesized by Gene Tools, LLC (Philomath, OR 97370 USA). MOs were prepared to 4 mM with sterile distilled water and stored in a humid environment at room temperature. The operation started at 8:30 a.m. on GD4. The mice were anesthetized first, and then 7.5 μL Cyp26a1 and Random Control MO solution were injected into the uterine horns of the treatment group and the control group, respectively. The mice were sacrificed on GD6 and the uterus on the side of MO injection was collected for further experiments.

### Cell Culture and Polarization of Macrophages

The immortalized bone-marrow-derived macrophages (iBMDM) cells were kindly provided by Dr. Shao F (National Institute of Biological Sciences, Beijing). iBMDM, HEK293T and Raw264.7 cells were grown in high-glucose Dulbecco’s modified Eagle’s medium (DMEM; Gibco, C11995500BT) supplemented with 10% heat-inactivated fetal bovine serum (FBS; Gibco, 10270-106) and 1% penicillin-streptomycin. Human monocyte THP-1 cells were cultured in Roswell Park Memorial Institute medium 1640 (RPMI 1640; Gibco, C11875500BT) supplemented with 10% heat-inactivated FBS, 1% penicillin-streptomycin and 50 pM β-mercaptoethanol. The bone-marrow-derived macrophages (BMDM) were collected from femur and tibia of female BALB/c mice (8–10 weeks of age), cultured in DMEM supplemented with 10% heat-inactivated FBS, 1% penicillin-streptomycin and 50 ng/mL M-CSF (BioLgend, 576402) for 7 days to generated macrophages under M0 state. Primary uterine macrophages sorted from GD6 mice labeled with CD45 (1:100, eBioscience, 11-0451-82) and F4/80 (1:200, eBioscience, 12-4801-80) were obtained by flow cytometry (FCM) and cultured in RPMI 1640 supplemented with 10% heat-inactivated FBS and 1% penicillin-streptomycin. All cells were grown in a humidified atmosphere containing 5% CO_2_ at 37°C.

THP-1 cells were treated with 160 nM phorbol 12-myristate 13-acetate (PMA; Sigma, P8139) for 24 h and a 24 h rest period in PMA-free medium to obtain the resting state of macrophages (M0). The resting macrophages were polarized into M1 state by incubation with 100 ng/mL LPS (Sigma, L3024) + 20 ng/mL IFN-γ (PeproTech, 300-02) or differentiated into M2 macrophages in the presence of 20 ng/mL IL-4 (PeproTech, 200-04) + 20 ng/mL IL-13 (PeproTech,200-13). iBMDM, Raw264.7 and BMDM cells were stimulated with 100 ng/mL LPS + 20 ng/mL INF-γ (PeproTech, 315-05) to differentiate into M1 or incubation with 20 ng/mL IL-4 (PeproTech, 214-14) + 20 ng/mL IL-13 (PeproTech, 210-13) to obtain M2 phenotype. Mouse CD45^+^F4/80^+^ uterine macrophages sorted from GD6 were seeded in 96-well plates at a density of 1 × 10^5^ cells/well and incubated with anti-CYP26A1 antibody (Ab) (1:25, Invitrogen, PA5-24602) or control IgG (1:50, Genscript, A01008) for 12 h, and then polarized into M1 or M2 state. The induction time was 4 h or 24 h.

### Generation of CYP26A1^−/−^ Raw264.7 Cells

CRISPR/Cas9-mediated gene knockout was performed as previously described with some minor adjustments (33). In brief, guide RNAs (gRNAs) targeting the exon1 or exon2 of *Cyp26a1* were designed using the CRISPR design website http://crispr.mit.edu. The gRNAs sequences were cloned into the pSpCas9 (BB)-2A-GFP (PX458) plasmid (Addgene, #48138) and then verified by sequencing. Raw264.7 cells (6.6 × 10^5^ cells/well) were seeded in 6-well plates the day before transfection to ensure that the cell density reached 70-90% prior to transfection. 2.5 μg gRNA-PX458 plasmid pair or PX458 vector were transfected into RAW264.7 cells with Lipofectamine LTX (Thermo, A12621) according to the manufacturers’ protocols. After 48 h, GFP-positive cells were sorted into 96-well plates by FCM. About two weeks later, growing monoclonal cells were first identified by genomic PCR genotyping and followed by DNA sequencing, q-PCR and Western Blot. CYP26A1^−/−^ Raw264.7 cells and empty vector wild-type (WT) were cryopreserved and used for further experiments. gRNAs sequences, PCR primers spanning gene deletion regions and qPCR primers partially within the deleted exon for clone identification in this study were listed in (**Table 1**).

**Table 1:** Primers used for CRISPR-Cas9-mediated CYP26A1 knockout in Raw264.7.

Primer name	Sequences (5’ - 3’)
Cyp26a1 gRNA upstream pair 1	Target: CACCGCCCTTGCCCCCCGGTACCAT Comp: AAACATGGTACCGGGGGGCAAGGGC
Cyp26a1 gRNA upstream pair 2	Target: CACCGGAGGGCGCAGCTGCGATCG Comp: AAACCGATCGCAGCTGCGCCCTCC
Cyp26a1 gRNA downstream pair 1	Target: CACCGAGGATGGTGCGCACCGACGC Comp: AAACGCGTCGGTGCGCACCATCCTC
Cyp26a1 gRNA downstream pair 2	Target: CACCGCGCCCATCACCCGCACCGT Comp: AAACACGGTGCGGGTGATGGGCGC
Primers used for PCR identification (966bp)	F: AGGGGCCCGATCCGCAATTA R: CGCCTTTCCGAGTACCCTTTCA
Primers 1 used for qPCR identification (218bp)	F: GCTCAAGCTCTGGGACCTGT R: CATTATCCGCGCCCATCACC
Primers 2 used for qPCR identification (144bp)	F: ACCTGTACTGTGTGAGCAGCC R: AGCCGTATTTCCTGCGCTTC

F, forward; R, reverse.

### Lentivirus Production and Infection

The CSII-EF-MCS-IRES2-Venus (RDB04384) lentiviral vector and packaging constructs, pCMV-VSV-G-RSV-Rev (RDB04393) and pCAG-HIVgp (RDB04394) were kindly provided by Dr. H. Miyoshi (RIKEN, BRC DNA Bank, Japan). In brief, Full-length of mouse *Cyp26a1* complementary DNAs amplified from GD6 mice uterine using PCR (Takara, R045Q) were inserted into the CSII-EF-MCS-IRES2-Venus vector (CSII-Vector) and then identified by genomic PCR genotyping and DNA sequencing. HEK293T cells were seeded at a density of 4 × 10^6^ cells in 10-cm culture dishes the day before transfection to ensure cells reach 90% confluence prior to transfection. 8 μg CSII-EF-MCS-IRES2-Venus carrying *Cyp26a1* (CSII-*Cyp26a1*) or CSII-Vector, and 8 μg packaging constructs were transfected into CYP26A1^−/−^ Raw264.7 cells with Lipofectamine 2000 (Thermo, 11668) and PEI (Polyscience, 23966) according to the manufacturers’ protocols. Lentivirus-containing culture medium was collected at 48 h and 72 h after transfection and passed through 0.45 μm cassette filters. The culture medium was centrifuged at 35 000 g for 2.5 h at 4°C to remove the supernatant. Lentivirus pellets were suspended with PBS and placed at 4°C overnight, and then stored at -80°C. CYP26A1^−/−^ Raw264.7 cells were infected with concentrated lentivirus containing 10 µg/ml polybrene (Beyotime, C0351). After 12 h, the medium was replaced with fresh DMEM complete medium and incubated for another 36 h. YFP positive cells were sorted by FCM. About 7 days later, growing cells were identified by genomic PCR, q-PCR and Western Blot. CSII-CYP26A1-CYP26A1^−/−^ Raw264.7 overexpression cells (CSII-KO-OE) and CSII-Vector-CYP26A1^−/−^ Raw264.7 cells (CSII-KO) were cryopreserved and used for further study. PCR and qPCR primers for plasmid construction, identification and overexpression cells identification in this study were listed in (**Table 2**).

**Table 2:** Primers used for lentivirus-mediated CYP26A1 overexpression in Raw264.7.

Primer name	Sequences (5’ - 3’)
Primers used for amplify Cyp26a1 (1520bp)	F: ATAAGAATGCGGCCGCCGTGCCATGGGGCTC-CCGGCGCTGCT R: CGGGATCCTCAGATATCTCCCTGGAAGTGG
Primers used for plasmid identification (1658bp)	F: CTCAAGCCTCAGACAGTGGT R: ACACCGGCCTTATTCCAAGC
Primers used for PCR identification (WT: 526bp; KO: 99bp; KO-OE: 99bp,334bp)	F: TCTGGGACCTGTACTGTGTGA R: TTCTTTCGCTGCTTGTGCG
Primers used for qPCR identification (218bp)	F: GCTCAAGCTCTGGGACCTGT R: CATTATCCGCGCCCATCACC

F, forward; R, reverse; WT, Raw264.7 wild-type cells; KO, Raw264.7 CYP26A1^−/−^ cells; KO-OE, CSII-CYP26A1-CYP26A1^−/−^ Raw264.7 overexpression cells.

### Cell Suspension Preparation and FCM

Cell preparation and FCM were performed as previously described with some minor modifications (34). In brief, uterine tissues were dissected with scissors to remove fat, mesangium and cervix. Then, these tissues were minced with scissors in 1640 medium containing 8% FBS, 1 mg/mL collagenase type IV (Sigma, C5138) and 0.3 mg/mL hyaluronidase (Sigma, H3506), and then enzymatically digested at 37°C, 160 rpm for 30 min. After digestion, cells were centrifuged at 1500 rpm for 5 min to remove the supernatant, and incubated in 1640 containing 2% FBS at 37°C, 160 rpm for 15 min prior to filtration through 400 mesh stainless steel cell strainer. The spleen tissues were minced carefully and filtered through 400 mesh stainless steel cell strainer. Cell lines cultured in 6-well or 12-well plates were detached with Trypsin−EDTA (0.25%) and washed with PBS, and then filtered through 200 mesh stainless steel cell strainer. Single-cell suspensions were blocked with anti-CD16/CD32 (1:100, eBioscience, 14-0161) for 15 min and then stained with fluorochrome-labeled antibody for 30 min. Antibodies used for staining included FITC anti-CD45 (1:100, eBioscience, 11-0451-82), PE anti-CD45 (1:200, eBioscience, 12-0451-81), PerCP-Cy5.5 anti-F4/80 (1:100, eBioscience, 45-4801-80), PE anti-F4/80 (1:200, eBioscience, 12-4801-80), APC anti-CD206 (1:30, R&D systems, FAB25351A), PE anti-CD86 (1:150, eBioscience, 12-0862-81). Cells were washed and suspended in PBS containing 2% FBS for FCM analysis using BD LSRFortessa or FACSAria IIIu (BD Biosciences). For sorting experiments, cells were sorted with BD AriaFusion (BD Biosciences). The FCM data were analyzed with Flowjo X 10.0.7 R2 software.

### Immunofluorescence

For live cells immunofluorescence, CD45^+^F4/80^+^CD206**^−^** M1-like and CD45^+^F4/80^+^CD206**^+^** M2-like macrophages sorted from GD6 mice uterine by FCM were incubated with anti-CYP26A1 antibody (1:50, Invitrogen, PA5-24602) for 1 h at 4°C. After washing, cells were incubated with fluorochrome-labeled secondary antibodies (1:200, Jackson ImmunoResearch Laboratories, 711-545-152) for 30 min, washed with PBS for twice times. Cells were then incubated with Hochest 33342 (10 μg/mL, Beyotime, c1022) at 37°C for 25 min. After washing, cell suspensions were dropped on glass slide and covered with cover glass for taking pictures. Images were acquired using Carl Zeiss LSM880 confocal microscope.

### Western Blot

Mice uterine tissues after cryogenic grinding or cell pellets were lysed in RIPA buffer (Cwbio, CW2333S) containing 1 mM PMSF (Sigma, 78830) on ice for 30 min before centrifuging at 4°C, 14 000 g for 15 min. The collected supernatants were quantified using BCA Protein Assay reagents (Pierce, 23227), then mixed with 5 × SDS loading buffer (Beyotime, p0015) and boiled for 10 minutes at 100°C. Proteins samples were separated by 10% SDS-PAGE and blotted onto nitrocellulose membranes (Pall, 66485). Membranes were blocked with 5% nonfat milk for 1 h at room temperature and then probed with the indicated primary antibodies for 1 h or overnight at 4°C. After washing thoroughly, the membranes were incubated with HRP-coupled secondary antibodies and visualized using Chemiluminescent Imaging System (Sagecreation, MiniChemi610). The images were analyzed with ImageJ software. Primary and secondary antibodies used for western blot were as follows: anti-CYP26A1 (1:1000, Abcam, ab151968), anti-Mannose Receptor (1:1000, Abcam, ab64693), anti-liver Arginase (1:1000, Abcam, ab60176), anti-GAPDH (1:1000, Cell signaling technology, 2118), HRP-conjugated goat anti-rabbit IgG (1:10000, Thermo, 31460) and HRP-conjugated bovine anti-goat IgG (1:5000, Jackson ImmunoResearch Laboratories, 805-035-180).

### Phagocytosis Assay

Raw264.7 cells (WT and CYP26A1**^−/−^**) phagocytic capacity was evaluated using fluorescence-labelled latex beads (Sigma, L3030). In brief, cells were seeded in 12-well plates at a density of 5 × 10^5^ cells/well and polarized to M1 or M2 state for 24 h. Then, the medium was replaced with 1 mL fresh FBS-free DMEM medium containing 4 μL red fluorescent latex beads and incubated for another 4 h. The supernatants were removed, and cells were washed with cool PBS for three times to remove excess beads. Subsequently, cells were detached with Trypsin−EDTA (0.25%) and washed with PBS. The phagocytosis of the cells was measured by FCM.

### Nitric Oxide (NO) Detection

The NO levels in supernatants of Raw264.7 cells (WT and CYP26A1**^−/−^**) were determined using NO assay kit (Beyotime, S0021) according to the manufacturers’ protocols. Briefly, cells were seeded in 96-well plates at a density of 2 × 10^4^ cells/well and polarized to M1 for 12 h, 24 h, 36 h, 48 h and 72 h. Subsequently, 50 μL of supernatants were collected and mixed with equal volumes of Griess reagent I and II in a new 96-well plate. The NO concentrations were determined at 540 nm using automatic microplate reader (Bio Tek, PowerWave XS).

### Cytokine Assays

Raw264.7 cells (WT, CYP26A1**^−/−^**, CSII-KO and CSII-KOOE) were seeded in 6-well plates at a density of 1.6 × 10^6^ cells/well and polarized into M1 state for 24 h. Then, the cell-free supernatants were collected and stored at -20°C for cytokine measurement. The concentrations of IL-1β, IL-6 and TNF-α secreted by Raw264.7 cells were determined using commercially enzyme-linked immunosorbent assay (ELISA) kit (R&D systems, MLB00C, M6000B and MTA00B) according to the manufacturers’ protocols.

### Histological Analysis

Mice uterine tissues were fixed with 4% paraformaldehyde at 4°C for 24 h and embedded in paraffin. Paraffin tissue sections were cut into 5 μm thick using paraffin microtome (Leica, RM2235) and stained with hematoxylin and eosin (H&E). Then the sections were observed with microscope (Nikon, ECLIPSE Ni) for histologic evaluation.

### RNA Extraction and Quantitative Real Time PCR (qPCR)

Total RNA was extracted from mice uterine tissues and cells using Trizol reagent (Invitrogen, 15596018), and then reversely transcribed into cDNA using M-MLV reverse transcriptase system (Promega, M1705) according to the manufacturer’s instructions. qPCR was performed using UltraSYBR Mixture (Cwbio, CW0957M) on a real-time PCR instrument (Roche, LightCycler 480 II). Cycle threshold (Ct) values of target gene were normalized to those of housekeeping gene *Gapdh* and 2^–ΔΔCt^ method was used to calculate relative abundance of gene expression between groups. The primer sequences used in the present study were provided in (**Supplementary Table S1**).

### RNA-Sequencing (RNA-Seq) Analysis

Raw264.7 cells (WT and CYP26A1**^−/−^**) were seeded in 6-well plates at a density of 1.6 × 10^6^ cells/well and polarized into M1 state for 4 h. Total RNA was extracted using Trizol reagent and RNA quality was evaluated using the Agilent 2100 Bioanalyzer and Agilent RNA 6000 Nano Kit. High-throughput sequencing of qualified cDNA library was performed using Illumina Novaseq 6000 platform (Annoroad Genomics). According to the sequencing results, the differentially expressed genes (DEGs) in WT and KO groups with two biological replicates were comprehensively analyzed. DESeq2 was used for gene differential expression analysis and genes with |log2 Fold change| ≥ 1and q < 0.05 were considered as DEGs. Gene Ontology (GO) and Kyoto Encyclopedia of Genes and Genomes (KEGG) analysis of DEGs were also performed.

### Statistical Analysis

The statistical analyses were performed with Excel 2010 and GraphPad Prism 8. All data were presented as the result of at least three independent experiments and expressed as the means ± SEM. Data between groups were analyzed by unpaired Student’s two-tailed t-test. *P < 0.05, **P < 0.01 and ***P < 0.001 were defined as statistically significant.

## Results

### Uterine M1-Like and M2-Like Macrophages Undergo Dynamic Changes During the Peri-Implantation Period

**Figure 1:**
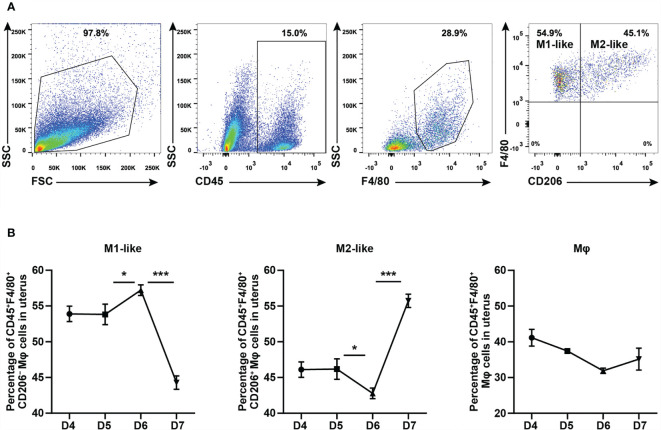
FCM analysis of dynamic changes of M1-like and M2-like uterine macrophages during the peri-implantation period in mice. **(A)** Gating strategy used to identify uterine M1-like macrophages (CD45^+^F4/80^+^CD206**^−^**) and M2-like macrophages (CD45^+^F4/80^+^CD206^+^) in pregnant mice. FSC *vs* SSC gating was used to clean up the debris and dead cells. CD45^+^ represents leukocyte; F4/80^+^ represents macrophages; CD206**^−^** and CD206^+^ represent M1-like and M2-like uterine macrophages, respectively. **(B)** Dynamic changes of M1-like macrophages, M2-like macrophages and Mφ during peri-implantation period in mice (n≥3). Error bars represent means ± SEM; two-tailed unpaired t-test, ns, not significant, *P < 0.05 or ***P < 0.001.

To explore whether the effect of CYP26A1 on embryo implantation is related to the imbalance of uterine macrophages, it is necessary to determine whether there is a dynamic change of uterine M1-like and M2-like macrophages during the peri-implantation period in mice. In this study, we used FCM to analyze the M1-like and M2-like subpopulations of uterine macrophages from GD4 to GD7. M1-like macrophages were marked as CD45^+^F4/80^+^CD206**^−^** and M2-like macrophages were stained as CD45^+^F4/80^+^CD206^+^ (**Figure 1A**). The results showed that the proportion of M1-like macrophages in F4/80^+^ macrophages significantly increased from GD5 to GD6, and then decreased rapidly from GD6 to GD7 (**Figure 1B**). The dynamic changes trend of M2-like macrophages was just opposite to that of M1-like macrophages (**Figure 1B**). There was no significant difference in the proportion of F4/80^+^ macrophages in CD45^+^ leukocytes from GD4 to GD7 (**Figure 1B**). Together, these results suggest that uterine M1-like and M2-like macrophages undergo dynamic changes during the peri-implantation period, and inflammatory uterine M1-like macrophages show an increasing trend in mice from GD5 to GD6, which may create an inflammatory environment to help embryos implant into the endometrium.

### CYP26A1 Was Differentially Expressed in M1 and M2 Macrophages

**Figure 2:**
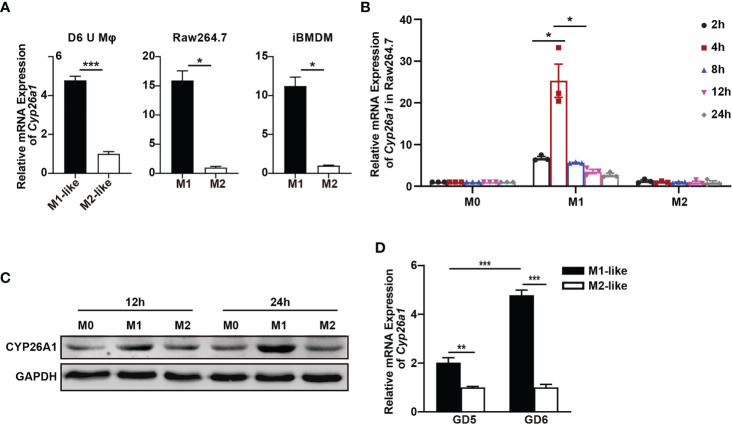
Differential expression of *Cyp26a1* in M1 and M2 macrophages. **(A)** qPCR analysis of *Cyp26a1* in M1 and M2 of GD6 uterine macrophages (D6 U Mφ), Raw264.7 and iBMDM (n=3). **(B)** qPCR analysis of *Cyp26a1* in M0, M1 and M2 macrophages of Raw264.7 at different induction time (LPS (100 ng/mL) + IFN-γ(20 ng/mL); 2 h, 4 h, 8 h, 12 h and 24 h; n=3). **(C)** Protein expression of CYP26A1 in M0, M1, M2 macrophages of Raw264.7 at different induction time (12 h and 24 h) was analyzed by Western blot. GAPDH was used as loading control. **(D)** qPCR analysis of *Cyp26a1* in M1-like and M2-like uterine macrophages of GD5 and GD6 (n=3). Error bars represent means ± SEM; two-tailed unpaired t-test, ns, not significant, *P < 0.05, **P < 0.01, or ***P < 0.001.

In order to investigate whether there is differential expression of *Cyp26a1* among different subtypes of macrophages, qPCR was used to detect *Cyp26a1* in M1 and M2 of GD6 uterine macrophages, GD6 spleen macrophages, BMDM and monocyte/macrophage cell lines (THP-1, Raw264.7 and iBMDM). We found that the expression of *Cyp26a1* was significantly upregulate in M1 as compared with M2 of GD6 uterine macrophages, Raw264.7 and iBMDM (**Figure 2A**). We did not detect the expression of *Cyp26a1* in M1 and M2 of BMDM, GD6 spleen macrophages and THP-1 (Ct values=35, data not shown). In addition, during the induction of RAW264.7 with LPS and IFN-γ, we found that the expression of *Cyp26a1* was different at different induction time (2 h, 4 h, 8 h, 12 h and 24 h). The expression of *Cyp26a1* reached the highest level at 4 h after induction, and this trend of changes was consistent with some inflammatory cytokines such as *Il1b* and *Il6* (**Figure 2B** and **Supplementary Figure 1**). In RAW264.7 cells, the protein level of CYP26A1 at different induction time (12 h and 24 h) was also detected by Western blot, and the results showed that the protein level of CYP26A1 in M1 was higher than that in M0 and M2 (**Figure 2C**). Moreover, the expression of *Cyp26a1* in GD6 uterine M1-like macrophages was significantly higher than that of GD5 M1-like macrophages (**Figure 2D**). Taken together, these results demonstrate that *Cyp26a1* was significantly upregulate in M1 as compared with M2 of GD6 uterine macrophages, Raw264.7 and iBMDM, which may indicate that CYP26A1 plays a certain role in the process of macrophages polarization.

### CYP26A1 Knockdown Disrupted the Balance of M1-Like and M2-Like Uterine Macrophages in Mice

**Figure 3:**
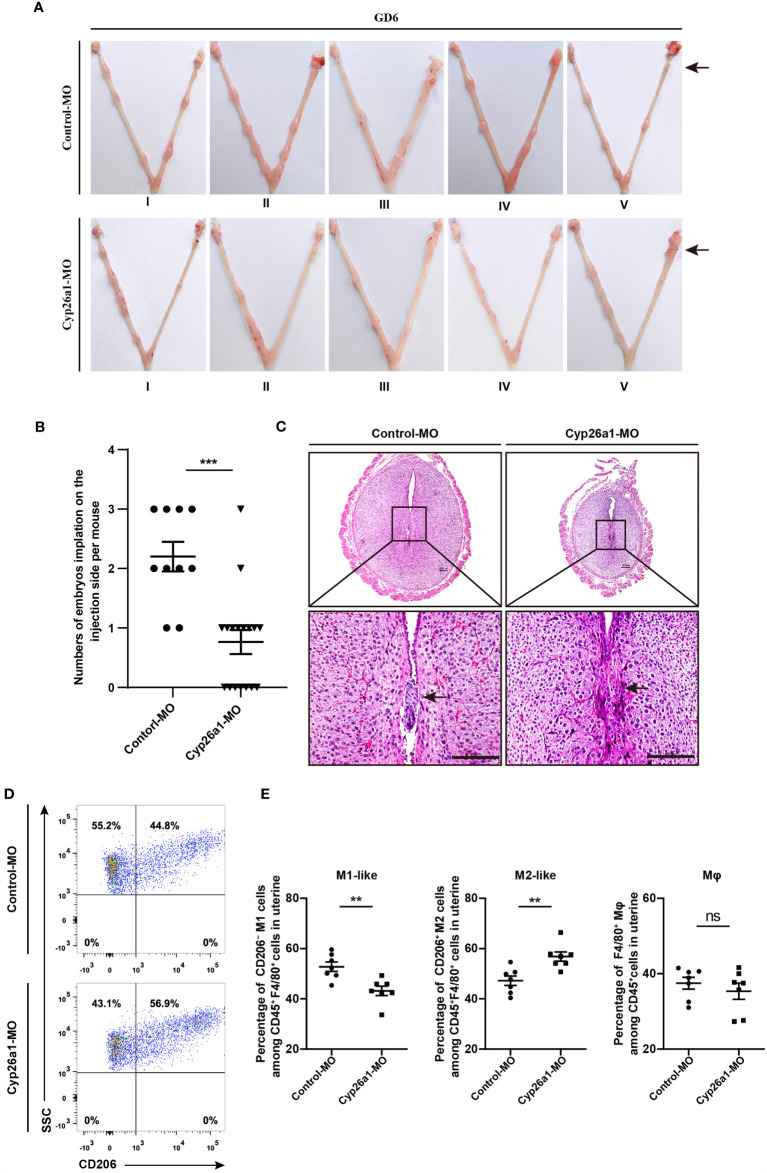
Knockdown of CYP26A1 in uterus affects embryo implantation and the balance of uterine M1-like and M2-like macrophages. **(A)** Representative macroscopic views of the uterus injected with Control-MO or Cyp26a1-MO. Arrows indicates the injection site. **(B)** Statistics of embryo implantation sites on the MO-injection side (Control-MO, n=10; Cyp26a1-MO, n=17). **(C)** H&E staining of tissue sections at the implantation sites. Scale bar, 100 µm. Arrows indicates the implantation sites. **(D, E)** FCM analysis of the ratio of uterine M1-like and M2-like macrophages on the MO-injection side (n=7). Error bars represent means ± SEM; two-tailed unpaired t-test, ns, not significant, **P < 0.01 or ***P < 0.001.

In our previous research, we found that CYP26A1 may affect embryo implantation by regulating NK and DC cells (30, 31). In addition, the disruption of macrophage M1/M2 balance can affect pregnancy outcome (7, 22). Here, we want to know whether the decrease of implantation sites caused by knockdown of CYP26A1 is related to the disruption of uterine M1-like and M2-like macrophages balance. We injected Cyp26a1-MO into the uterine horn of mice on GD4 to inhibit the translation of *Cyp26a1* mRNA and terminated the pregnancy on GD6 for FCM analysis. The results showed that the number of implantation sites was significantly reduced as compared to controls (**Figures 3A, B**). H&E staining of tissue sections at the implantation site in the treatment group could observe obvious structural and morphological abnormalities, including the reduction of the whole embryo ball, abnormal egg column and disappearance of the yolk sac cavity (**Figure 3C**). Moreover, we detected the proportion of CD45^+^F4/80^+^CD206**^−^** M1-like and CD45^+^F4/80^+^CD206^+^ M2-like macrophages in uterus by FCM. The results indicated that the proportion of M1-like macrophages significantly decreased, and the proportion of M2-like macrophages significantly increased in the treatment group as compared with the control group (**Figures 3D, E**). Knockdown of CYP26A1 had no effect on total uterine macrophages (**Figure 3E**). Taken together, these results suggest that the specific expression of CYP26A1 during the peri-implantation period may be involved in regulating the differentiation of uterine macrophages into M1-like subtype, which participates in the establishment of an inflammatory environment during embryo implantation. Knockdown of CYP26A1 reduces the proportion of M1-like macrophages in the uterus, resulting in insufficient inflammation and failed embryo implantation.

### Inhibiting the Activity of CYP26A1 Down Regulated the Polarization Level of M1 Uterine Macrophages

**Figure 4:**
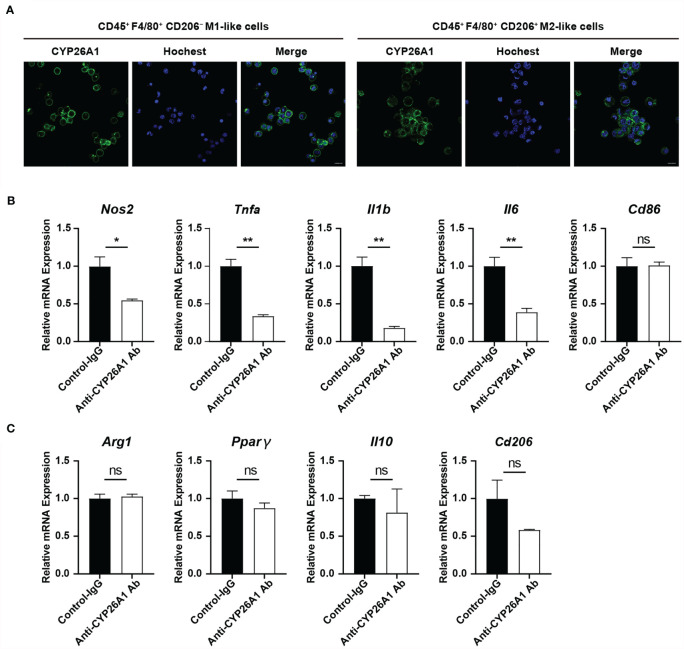
The effect of inhibiting the activity of CYP26A1 on the polarization level of uterine macrophages. **(A)** Live cells immunofluorescence analysis CYP26A1 expression in M1-like and M2-like macrophages isolated from the uterus on GD6 mice. Scale bar, 10 µm. **(B)** qPCR analysis of M1 markers (*Nos2*, *Tnfa*, *Il1b* and *Il6*) on uterine macrophages treated with anti-CYP26A1 Ab or control IgG for 12 h and then induced with LPS and IFN-γ for 4 h (n=3). **(C)** qPCR analysis of M2 markers (*Arg1*, *Pparg*, *Il10* and *Cd206*) on uterine macrophages treated with anti-CYP26A1 Ab or control IgG for 12 h and then induced with IL-4 and IL-13 for 4 h (n=3). Error bars represent means ± SEM; two-tailed unpaired t-test, ns, not significant, *P < 0.05 or **P < 0.01.

To further explore the effect of CYP26A1 on the polarization of macrophages, we used FCM to isolate mouse uterine macrophages on GD6 for polarization studies. Our previous studies have found that CYP26A1 is expressed on the membrane of NK cells and 8-cell embryos (28, 35). In this study, we performed live cell immunofluorescence to detect the expression of CYP26A1 in uterine M1-like and M2-like macrophages sorted from GD6 mice uterine. Live-cell immunofluorescence showed that CYP26A1 was mainly localized in the cell membrane of uterine M1-like and M2-like macrophages (**Figure 4A** and **Supplementary Figure 2**). Subsequently, we isolated CD45^+^F4/80^+^ uterine macrophages and treated them with anti-CYP26A1 Ab, and then added M1 or M2 inducers for 4 h to detect related polarization molecules. We found that the expression of M1 markers *Nos2*, *Tnfa*, *Il1b* and *Il6* was significantly decreased in anti-CYP26A1 Ab treated group compared with control-IgG group (**Figure 4B**). However, anti-CYP26A1 Ab treatment did not affect the expression of M2 markers *Arg1*, *Pparg*, *Il10* and *Cd206* (**Figure 4C**). These results suggest that inhibition of CYP26A1 activity down regulates the polarization level of uterine M1 macrophages but has no effect on uterine M2 macrophages.

### CYP26A1 Knockout Affected the Polarization and Function of Raw264.7 Cells

**Figure 5:**
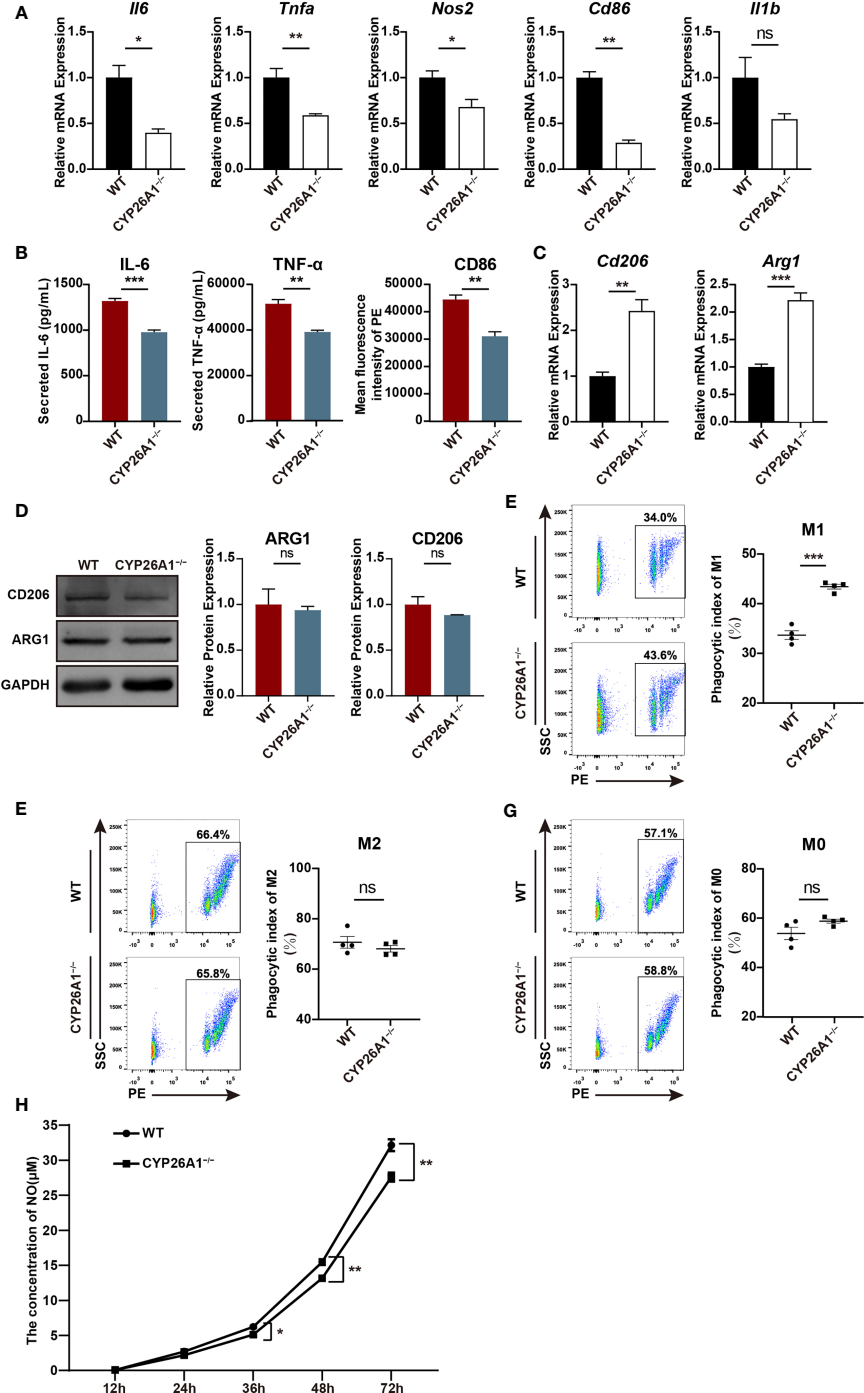
CYP26A1 deficiency affected the polarization, phagocytic capacity and NO production capacity of Raw264.7 cells. **(A)** qPCR analysis of M1 phenotype genes *Il6*, *Nos2*, *Tnfa*, *Cd86* and *Il1b* in CYP26A1-deficient Raw264.7 and WT cells after induced 4 h by LPS and IFN-γ (n=3). **(B)** ELISA was used to measure IL-6 and TNF-α secretion in supernatants of Raw264.7 cells (WT and CYP26A1^−/−^) treated with LPS and IFN-γ for 24 h; Mean fluorescence intensity analysis of CD86 expression levels in Raw264.7 cells (WT and CYP26A1^−/−^) incubation with LPS and IFN-γ for 24 h (n=3). **(C)** qPCR analysis of M2 markers *Arg1* and *Cd206* in CYP26A1 knockout Raw264.7 and WT group after induced 4 h by IL-4 and IL-13 (n=3). **(D)** Western blot analysis of M2 markers ARG1 and CD206 in CYP26A1 knockout Raw264.7 and WT group after induced 24 h by IL-4 and IL-13 (n=3). GAPDH was used as loading control. **(E–G)** Flow cytometry analysis of the phagocytic capacity of Raw264.7 (WT and CYP26A1^−/−^) under M0, M1 and M2 state (n=4). **(H)** The concentration of NO in the supernatant of RAW264.7 cells (WT and CYP26A1^−/−^) treated with LPS and IFN-γ at different induction time (12 h, 24 h, 36 h, 48 h, 72 h) was detected by griess reagent (n=4). Error bars represent means ± SEM; two-tailed unpaired t-test, ns, not significant, *P < 0.05, **P < 0.01, or ***P < 0.001.

To further investigate the role of CYP26A1 in macrophages polarization and function, we generated CYP26A1 knockout Raw264.7 using CRISPR/Cas9-mediated gene editing (**Supplementary Figure 3A**). CYP26A1 knockout cells were identified by genomic PCR, qPCR and Western blot (**Supplementary Figures 3B–D**). Compared with control group, CYP26A1^−/−^ Raw264.7 cells expressed significantly lower levels of M1 markers *Il6*, *Tnfa*, *Nos2* and *Cd86* after treated with LPS and IFN-γ for 4 h (**Figure 5A**). The protein levels of IL-6, TNF-α and CD86 in CYP26A1^−/−^ Raw264.7 cells also significantly decreased as compared with wild type (WT) after treated with LPS and IFN-γ for 24 h (**Figure 5B**). In addition, compared to WT cells, the mRNA expression of M2 markers *Arg1* and *Cd206* was significantly increased in CYP26A1^−/−^ Raw264.7 cells after treated with IL-4 and IL-13 for 4 h (**Figure 5C**). However, there was no significant difference in the protein levels of ARG1 and CD206 between two groups (**Figure 5D**). Moreover, we also performed phagocytosis assay and NO assay to study the effect of CYP26A1 knockout on macrophages function. The results indicated that CYP26A1-deficient Raw264.7 showed increased phagocytic activity under M1 stimulation state compared with control cells (**Figure 5E**). But this phenomenon was not found in M0 and M2 state (**Figures 5F, G**). The NO assay results showed that after 36 h of stimulation by LPS and IFN-γ, the NO production capacity of CYP26A1-deficient cells was significantly lower than that of the control group (**Figure 5H**). Taken together, these results suggest that CYP26A1 knockout can affect the polarization, phagocytic capacity and NO production capacity in Raw264.7 cells.

### Re-Introduction of CYP26A1 Partially Reversed the Polarization Levels of M1 in CYP26A1^−/−^ Raw264.7 Cells

**Figure 6:**
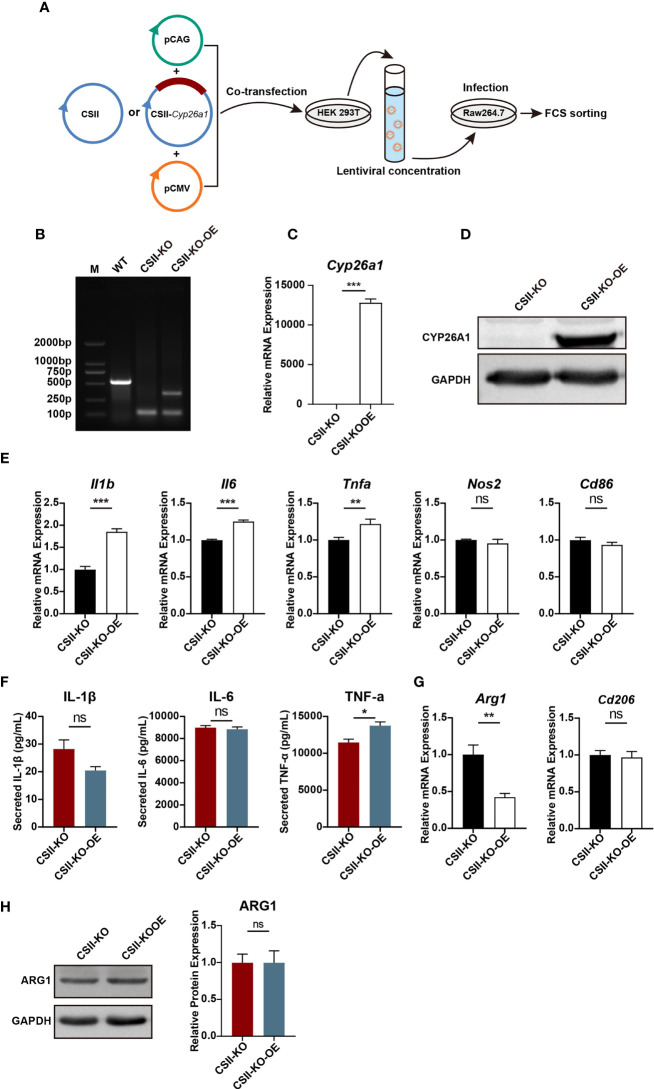
Re-introduction of CYP26A1 partially reversed the polarization levels of M1 in CYP26A1^−/−^ Raw264.7 cells. **(A)** Schematic diagram of CYP26A1 overexpression in CYP26A1^−/−^ Raw264.7 cells with the lentiviral expression vector. **(B–D)** Genomic PCR, qPCR and Western blot were used to identify CYP26A1 overexpression cells. PCR products, WT: 526bp; KO: 99bp; KO-OE: 99bp, 334bp. **(E)** qPCR analysis of M1 phenotype genes *Il1b*, *Il6*, *Tnfa*, *Nos2* and *Cd86* in CSII-KO and CSII-KO-OE cells after induced 4 h by LPS and IFN-γ (n=3). **(F)** ELISA was used to measure IL-1β, IL-6 and TNF-α secretion in supernatants of Raw264.7 cells (CSII-KO and CSII-KO-OE) treated with LPS and IFN-γ for 24 h (n=3). **(G)** qPCR analysis of M2 markers *Arg1* and *Cd206* in Raw264.7 cells (CSII-KO and CSII-KO-OE) after induced 4 h by IL-4 and IL-13 (n=3). **(H)** Western blot analysis of M2 markers ARG1 in CSII-KO and CSII-KO-OE cells after induced 24 h by IL-4 and IL-13 (n=3). GAPDH was used as loading control. Error bars represent means ± SEM; two-tailed unpaired t-test, ns, not significant, *P < 0.05, **P < 0.01, or ***P < 0.001.

To further investigate the role of CYP26A1 in macrophages polarization, we overexpressed CYP26A1 in CYP26A1^−/−^ Raw264.7 cells with the lentiviral expression vector (**Figure 6A**). We detected the transfection efficiency of HEK 293T cells by fluorescence microscopy and the percentage of YFP-positive RAW264.7 cells through FCM (**Supplementary Figure 4**). Genomic PCR, qPCR and Western blot were used to identify YFP-positive RAW264.7 cells sorted by FCM (**Figures 6B–D**). We found that CSII-KO-OE cells expressed significantly higher levels of M1 markers *Il1b*, *Il6* and *Tnfa* than that of the CSII-KO cells after treated with LPS and IFN-γ for 4 h (**Figure 6E**). The protein level of TNF-α in CSII-KO-OE cells significantly increased as compared with CSII-KO cells after treated with LPS and IFN-γ for 24 h (**Figure 6F**). In addition, compared with CSII-KO cells, the mRNA expression of M2 marker *Arg1* was significantly decreased in CSII-KO-OE cells after treated with IL-4 and IL-13 for 4 h (**Figure 6G**). However, there was no significant difference in the protein level of ARG1 between CSII-KO and CSII-KO-OE cells (**Figure 6H**). Together, these results suggest that overexpression of CYP26A1 partially reverse the polarization levels of M1 in CYP26A1^−/−^ Raw264.7 cells.

### CYP26A1 Knockout Changed the Transcription Profiles of Raw264.7 Cells

**Figure 7:**
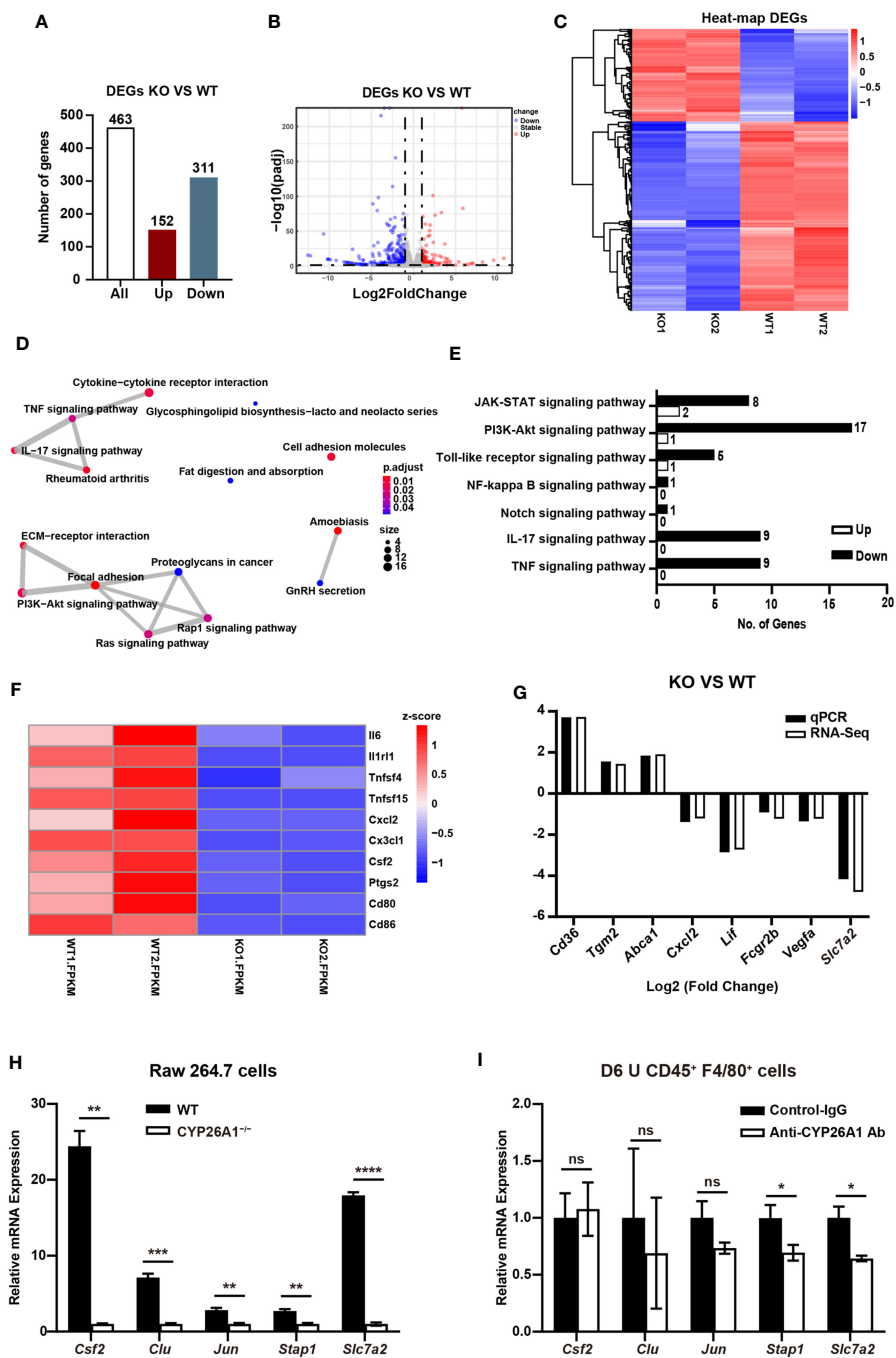
CYP26A1 knockout changed the transcription profiles of Raw264.7 cells **(A)** The number of genes that are significantly different between the KO and WT groups. **(B)** Volcano plot showing up-regulated genes (red dots) and down-regulated genes (blue dots). **(C)** Hierarchical clustering demonstrating a distinguishable genes expression pattern between WT and KO groups. **(D)** Genes overlap relationship analysis on the top 15 pathways enriched by KEGG **(E)** KEGG analysis of the inflammatory related pathway. **(F)** Z-scores of genes associated with inflammation in DGEs. **(G)** Comparison of validated qPCR assays with RNA-Seq for selected DEGs. **(H)** qPCR analysis of *Csf2*, *Clu*, *Jun*, *Stap1* and *Slc7a2* in CYP26A1-deficient Raw264.7 and WT cells after induced 4 h by LPS and IFN-γ (n=3). **(I)** qPCR analysis of *Csf2*, *Clu*, *Jun*, *Stap1* and *Slc7a2* in primary uterine macrophages treated with anti-CYP26A1 Ab or Control-IgG for 12 h and then induced with LPS and IFN-γ for 4 h (n=3). Error bars represent means ± SEM; two-tailed unpaired t-test, ns, not significant, *P < 0.05, **P < 0.01, ***P < 0.001 or ****P < 0.0001.

In order to conduct a more comprehensive assessment of the effect of CYP26A1 on macrophages. We performed global transcriptome analysis through RNA-Seq in CYP26A1**^−/−^** (KO) and WT Raw264.7 cells treated with LPS and IFN-γ for 4 h. Sequencing results showed that there were 463 DEGs between KO and WT groups (Fold change ≥ 2; P value ≤ 0.05), including 152 upregulated genes and 311 downregulated genes (**Figure 7A**). Volcano plot and hierarchical clustering demonstrated that KO group had different gene expression patterns compared with the WT group (**Figures 7B, C**). We performed gene overlap relationship analysis on the top 15 pathways enriched by KEGG (**Figure 7D**). In addition, the top 36 significantly enriched KEGG pathways were also listed in (**Supplementary Figure 5A**). The top 5 enriched GO terms were illustrated in (**Supplementary Figures 5B–D**). We found that the expression of some inflammatory and inflammatory signaling pathway-related genes decreased significantly in KO cells compared with WT cells (**Figures 7E, F**). We further picked some genes related to inflammation (*Cxcl2*, *Lif* and *Fcgr2b*), phagocytosis (*Cd36*, *Tgm2* and *Abca1*), NO production (*Slc7a2*) and angiogenesis (*Vegfa*) for qPCR identification to verify the reliability of the sequencing results (**Supplementary Figure 5E**). The qPCR results were consistent with the RNA-Seq results (**Figure 7G**). These results suggest that compared with the control group, knockout of CYP26A1 down-regulated the expression of some inflammation-related genes in Raw264.7 cells. In addition, the expression of some genes related to macrophage phagocytosis and NO production has also changed.

In order to further make a preliminary exploration of related molecules that may be involved in CYP26A1 regulating the polarization of uterine macrophages to M1. We verified the genes related to macrophages polarization in DEGs from biological processes (BP) in GO by qPCR in M1 state of Raw264.7 cells and primary uterine macrophages. *Csf2*, *Clu*, *Jun*, *Stap1* and *Slc7a2* have been reported to be involved in the polarization of macrophages (36–40). In Raw264.7 cells, we found that compared with control cells, the mRNA expression of *Csf2*, *Clu*, *Jun*, *Stap1* and *Slc7a2* was significantly decreased in CYP26A1^−/−^ Raw264.7 cells (**Figure 7H**). The qPCR results were consistent with the RNA-Seq results (**Supplement Figures 5F, G**). In CD45^+^F4/80^+^ primary uterine macrophages, the result indicated that the expression of *Stap1* and *Slc7a2* was significantly decreased in anti-CYP26A1 Ab treated group compared with control-IgG group, but there was no significant difference in the expression of *Csf2*, *Clu* and *Jun* (**Figure 7I**). These results preliminarily indicate that CYP26A1 may regulate the polarization of uterine macrophages to M1 through *Slc7a2* and *Stap1*.

## Discussion

CYP26A1 has been confirmed to play important roles in embryonic development and body patterning. In zebrafish, mutants lacking CYP26A1 display developmental defects in the hindbrain, spinal cord and tail (41). CYP26A1 knockout mice largely die during mid-late gestation and show morphogenetic defects, including spina bifida, caudal agenesis and hindbrain misspatterning (25, 26). In humans, the loss of CYP26A1 function may be related to spina bifida (42). Subsequent studies confirmed that CYP26A1 may affect embryonic development by degrading retinoic acid (RA) (43, 44). Recent studies have shown that knockout of CYP26A1 in juvenile or adult mice does not cause apparent retinoid toxicity and has no effect on their survival and health (45). This finding suggests that CYP26A1 has a minor role in modulating endogenous RA homeostasis in postnatal life, or that the function of CYP26A1 compensated by other molecules.

In our previous study, we have found that both CYP26A1 mRNA and protein have a specific expression pattern in mice uterine during blastocyst implantation period, and knockdown of CYP26A1 can significantly reduce the number of implantation sites (29). Further studies have found that there is no significant difference in the level of all-trans-retinoic acid (at-RA, the primary biologically active isomer of RA) in the uterus of mice from GD4 to GD7 by high performance liquid chromatography (data not published). In addition, we also have found that the concentration of at-RA has not significant change after knockdown of CYP26A1, and intraperitoneal injection of at-RA don’t affect embryo implantation in mice (data not published). In another study, at-RA has been found not to be involved in regulating embryo implantation in rats (46). Based on these results, we speculate that CYP26A1 may affect embryo implantation into the endometrium mainly through RA metabolic during the peri-implantation period in mice. In recent studies, we have found that CYP26A1 may regulate NK cells through chemokines during the peri-implantation period (30). In addition, we also have found that CYP26A1 can affect embryo implantation by regulating DC cells through ID2 and CD86 (31). These results indicate that CYP26A1 may affect mouse embryo implantation through regulating maternal immune cells.

The activation state of uterine M1/M2 macrophages presents dynamic changes during normal pregnancy, and inappropriate polarization of uterine macrophages can cause adverse pregnancy outcomes (21). Therefore, we want to study whether the effect of CYP26A1 on embryo implantation is related to the imbalance of uterine macrophages. In this study, we first detected the expression of *Cyp26a1* in several different types of macrophages and we found that this gene was significantly upregulated in M1 as compared with M2 of GD6 uterine macrophages, Raw264.7 and iBMDM. The RNA-seq of uterine macrophages in another study also showed that the expression of *Cyp26a1* in M1 was significantly higher than that of M2 in mice (47). However, it should be noted that we did not detect the expression of *Cyp26a1* in M1 and M2 of BMDM, GD6 spleen macrophages and THP-1. We speculated that this result may be due to the heterogeneity and highly different transcriptional profiles of macrophages in different tissues (48, 49). Subsequently, we confirmed that the expression of *Cyp26a1* was different during the induction of RAW264.7 with LPS and IFN-γ at different time and this trend of changes was consistent with some inflammatory cytokines such as *Il1b* and *Il6*. Based on these findings, we speculate that *Cyp26a1* may play an important role in the polarization of macrophages to the M1 subtype. And then, we used flow cytometry to detect the M1-like and M2-like uterine macrophages form GD4 to GD7, and we found that the proportion of M1-like and M2-like uterine macrophages changed dynamically during the peri-implantation period. Interestingly, the expression of *Cyp26a1* in GD6 uterine M1-like macrophages was significantly higher than that of GD5 M1-like macrophages. This result indicates that the expression of *Cyp26a1* was also different in uterine M1-like macrophages in different gestation days. Subsequently, we chose GD6 to further study the relationship between CYP26A1 and macrophage polarization.

We used the Cyp26a1-MO knockdown mice model to study the relationship between CYP26A1 and macrophages. Flow cytometry results showed that the proportion of CD45^+^F4/80^+^CD206^−^ M1-like macrophages decreased and the proportion of CD45^+^F4/80^+^CD206^+^ M2-like macrophages increased in Cyp26a1-MO mice compared with the control group. During embryo implantation, trophoblast cells have to penetrate uterine epithelial cells and stromal cells (50). The characteristics of this process are pro-inflammatory response in which high levels of pro-inflammatory cytokines are involved (50–53). Clinical studies have also confirmed that injury-induced inflammation can promote embryo implantation (54–56). Herein, our results indicated that the specific expression of CYP26A1 during the peri-implantation period may be involved in regulating the differentiation of uterine macrophages into M1 subtype, which participated in the establishment of inflammatory response microenvironment. Inhibiting the expression of CYP26A1 in the uterus resulted in insufficient inflammation response and failed embryo implantation.

The results in primary uterine macrophages and cell lines further confirmed that CYP26A1 could regulate the polarization of macrophages towards M1 phenotype. In addition, CYP26A1 knockout can affect the phagocytic capacity and NO production capacity in Raw264.7 cells. RNA-Seq analysis of CYP26A1 knockout Raw264.7 also showed that the expression of some inflammation and function related genes had changed. Moreover, RNA-Seq results showed that some DEGs are enriched in inflammation signaling pathways. These inflammation-related signaling pathways that may be regulated by CYP26A1 deserve to be further investigated. In this study, we preliminarily explored the relevant molecules that may be involved in the regulation of macrophage polarization to M1 by CYP26A1. We found that the expressions of *Slc7a2* and *Stap1* decreased significantly in CYP26A1 knockout Raw264.7 and anti-CYP26A1 Ab treated primary uterine macrophages. *Slc7a2* and *Stap1* have been confirmed to be involved in the polarization of macrophages towards M1 (39, 40). Therefore, we preliminarily speculate that CYP26A1 may regulate the polarization of uterine macrophages to M1 through *Slc7a2* and *Stap1*. But the more convincing results need to be further studied.

**Figure 8:**
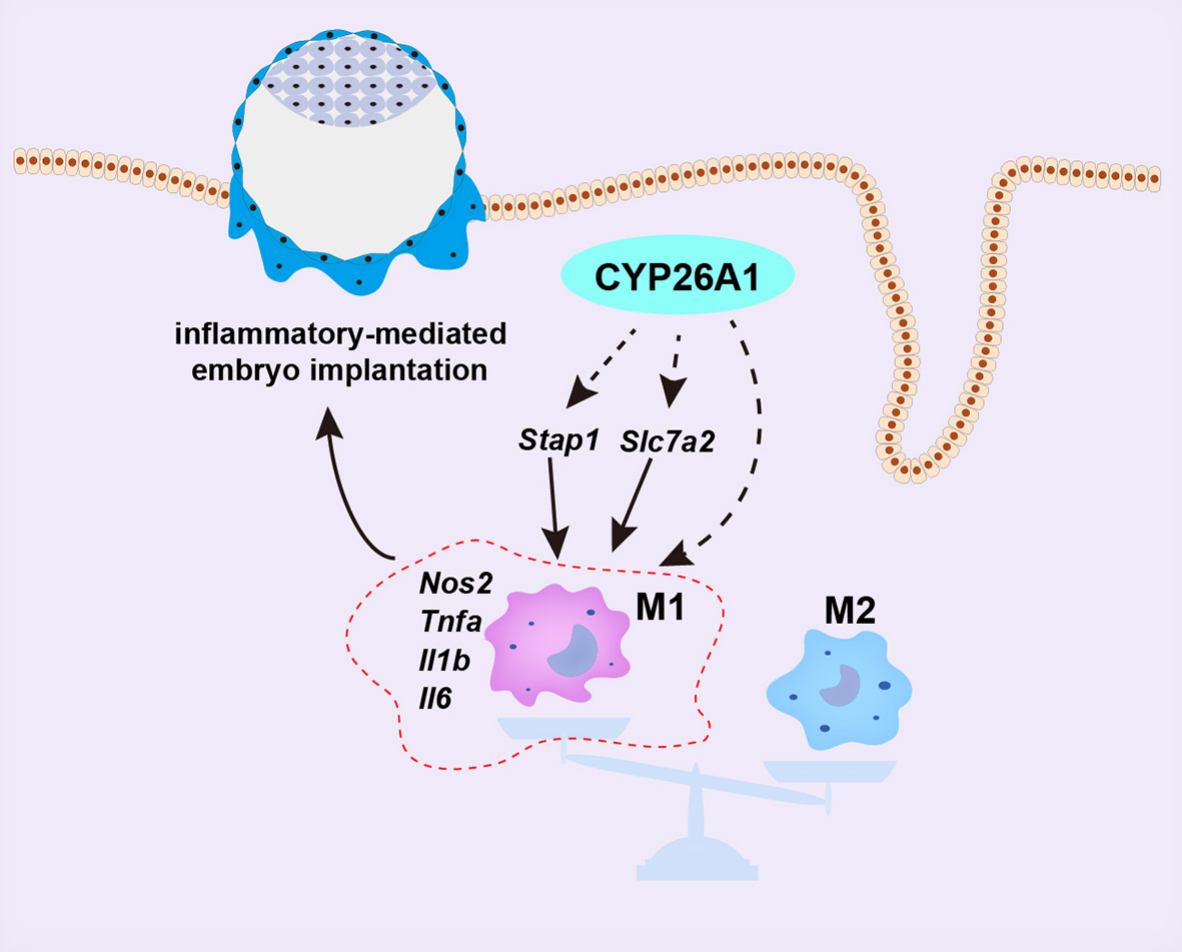
Schematic representation of CYP26A1 affects embryo implantation by regulating uterine macrophage polarization. During the peri-implantation period, CYP26A1 polarizes uterine macrophages towards M1 to provide a certain inflammatory response for embryo implantation. Knockdown of CYP26A1 caused insufficient M1 polarization, resulting in insufficient inflammation and failed embryo implantation. CYP26A1 may regulate the polarization of uterine macrophages to M1 through *Stap1* and *Slc7a2*.

In conclusion, our data demonstrate that the specific expression of CYP26A1 during the peri-implantation period may be involve in regulating the differentiation of uterine macrophages into M1 subtype, which participates in the establishment of an inflammatory environment during embryo implantation (**Figure 8**). Knockdown of CYP26A1 reduces the proportion of M1 macrophages in the uterus, resulting in insufficient inflammation and failed embryo implantation. This is a novel mechanism in which CYP26A1 can affect embryo implantation by regulating uterine macrophages polarization. Our results provide new evidence that the mother needs a certain inflammatory response during the peri-implantation period to promote embryo implantation, and also provide a new perspective for understanding the complex maternal immune regulation during pregnancy.

## Data Availability Statement

The original contributions presented in the study are publicly available. This data can be found here: https://www.ncbi.nlm.nih.gov/sra/PRJNA758264.

## Ethics Statement

The animal study was reviewed and approved by the ethical guidelines of the Animal Care and Use Committee of Institute of Zoology, Chinese Academy of Sciences.

## Author Contributions

J-PP conceived and designed the research, supervised the overall experiment process, and reviewed the final manuscript. W-HJ designed and performed the experiment, analyzed the data, and wrote the manuscript. D-DL performed the experiment and analyzed the data. D-PW provided critical technical support and contributed to data analysis. A-QG and YY provided critical technical support. All authors contributed to the article and approved the submitted version.

## Funding

This work was supported by grants from the National Natural Science Foundation of China (no. 31571552).

## Conflict of Interest

The authors declare that the research was conducted in the absence of any commercial or financial relationships that could be construed as a potential conflict of interest.

## Publisher’s Note

All claims expressed in this article are solely those of the authors and do not necessarily represent those of their affiliated organizations, or those of the publisher, the editors and the reviewers. Any product that may be evaluated in this article, or claim that may be made by its manufacturer, is not guaranteed or endorsed by the publisher.
