# Supplementary material for: Cytochrome P450 26A1 Modulates the Polarization of Uterine Macrophages During the Peri-Implantation Period
Source: Front Immunol. 2021 Oct 12;12:763067. doi: 10.3389/fimmu.2021.763067 (PMC8546204; doi:10.3389/fimmu.2021.763067)
Supplement: Supplementary file 1 [file DataSheet_1.docx]

Supplementary Material


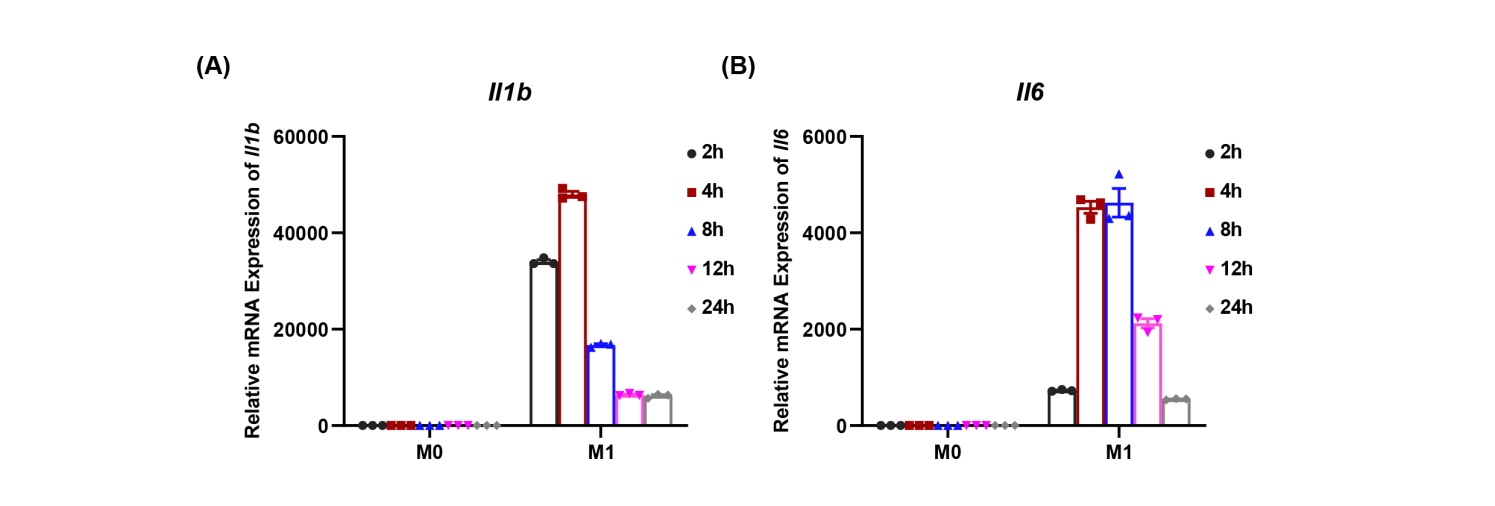


**Supplementary Figure 1** qPCR analysis of *Il1b* and *Il6* in M0 and M1 macrophages of Raw264.7 at different induction time (LPS (100 ng/mL) + IFN-γ(20 ng/mL); 2 h, 4 h, 8 h, 12 h and 24 h; n=3).


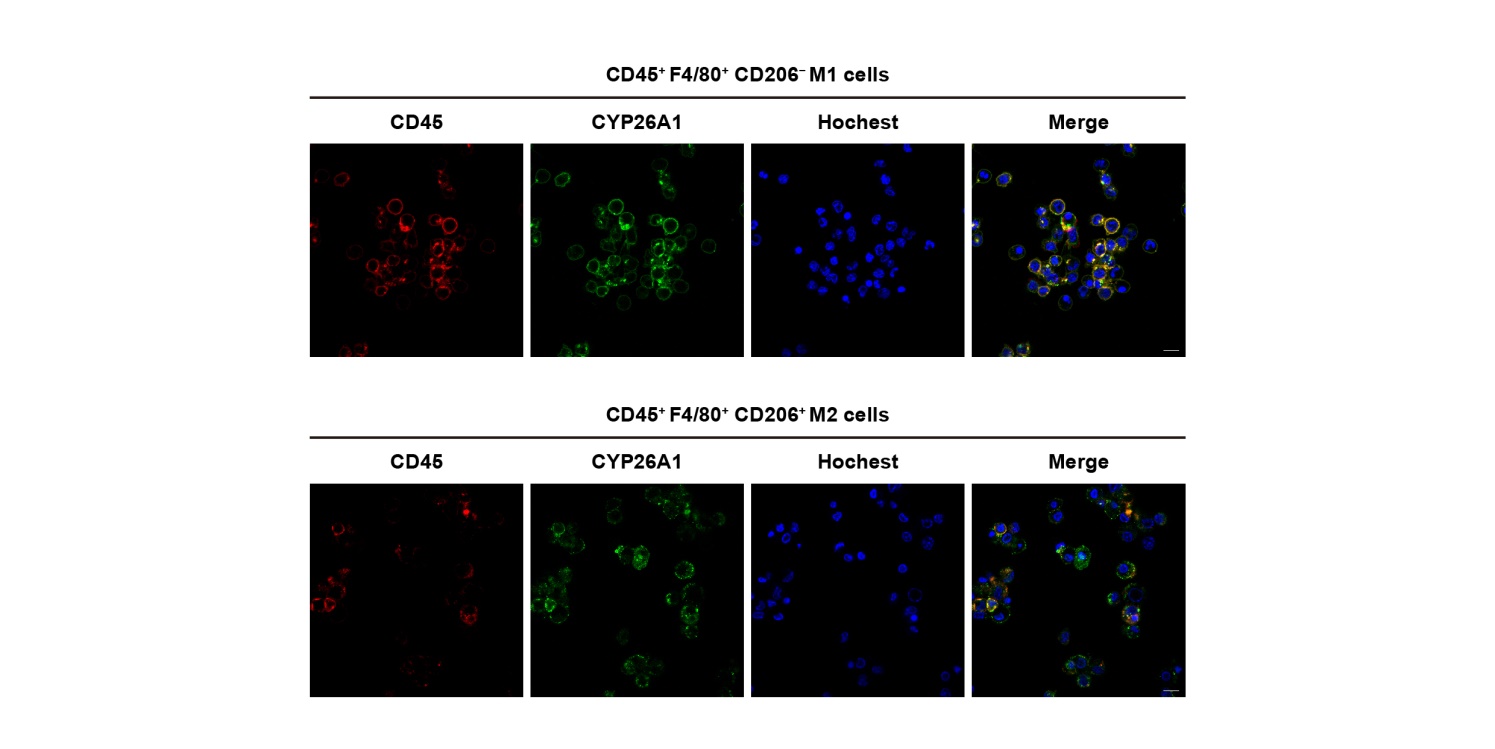


**Supplementary Figure 2** Immunofluorescence co-localization of CD45 and CYP26A1 within CD45^+^F4/80^+^CD206**^−^** M1-like and CD45^+^F4/80^+^CD206^+^ M2-like macrophages isolated from the uterus on GD6 mice. Scale bar, 10 µm.


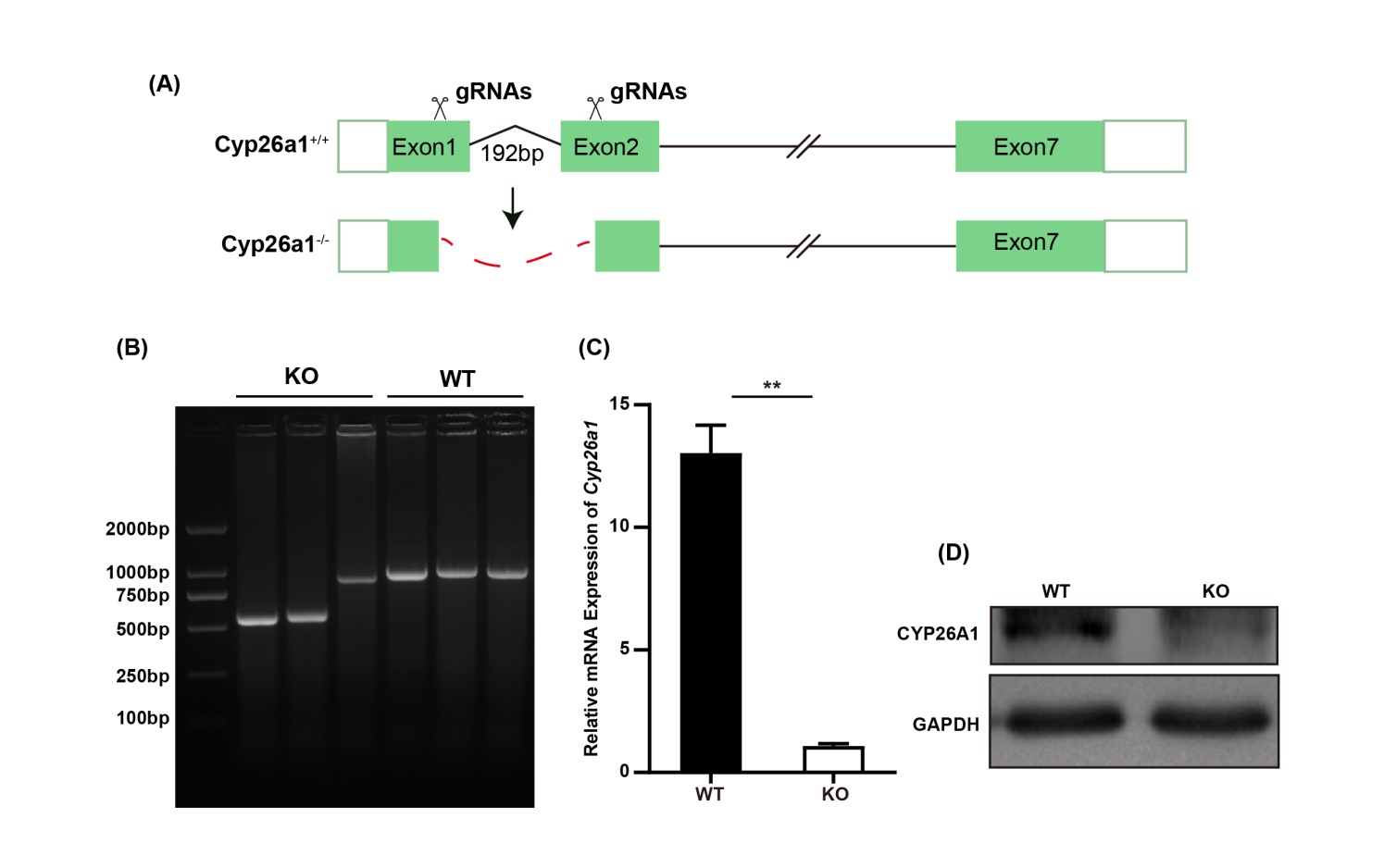


**Supplementary Figure 3** CRISPR/Cas9 mediated knockout and identification of CYP26A1 in Raw264.7 cells. **(A)** Schematic diagram of CYP26A1 knockout in CYP26A1^−/−^ Raw264.7 cells using CRISPR/Cas9. **(B)** Genomic PCR, qPCR and Western blot were used to identify CYP26A1 knockout Raw264.7 cells.


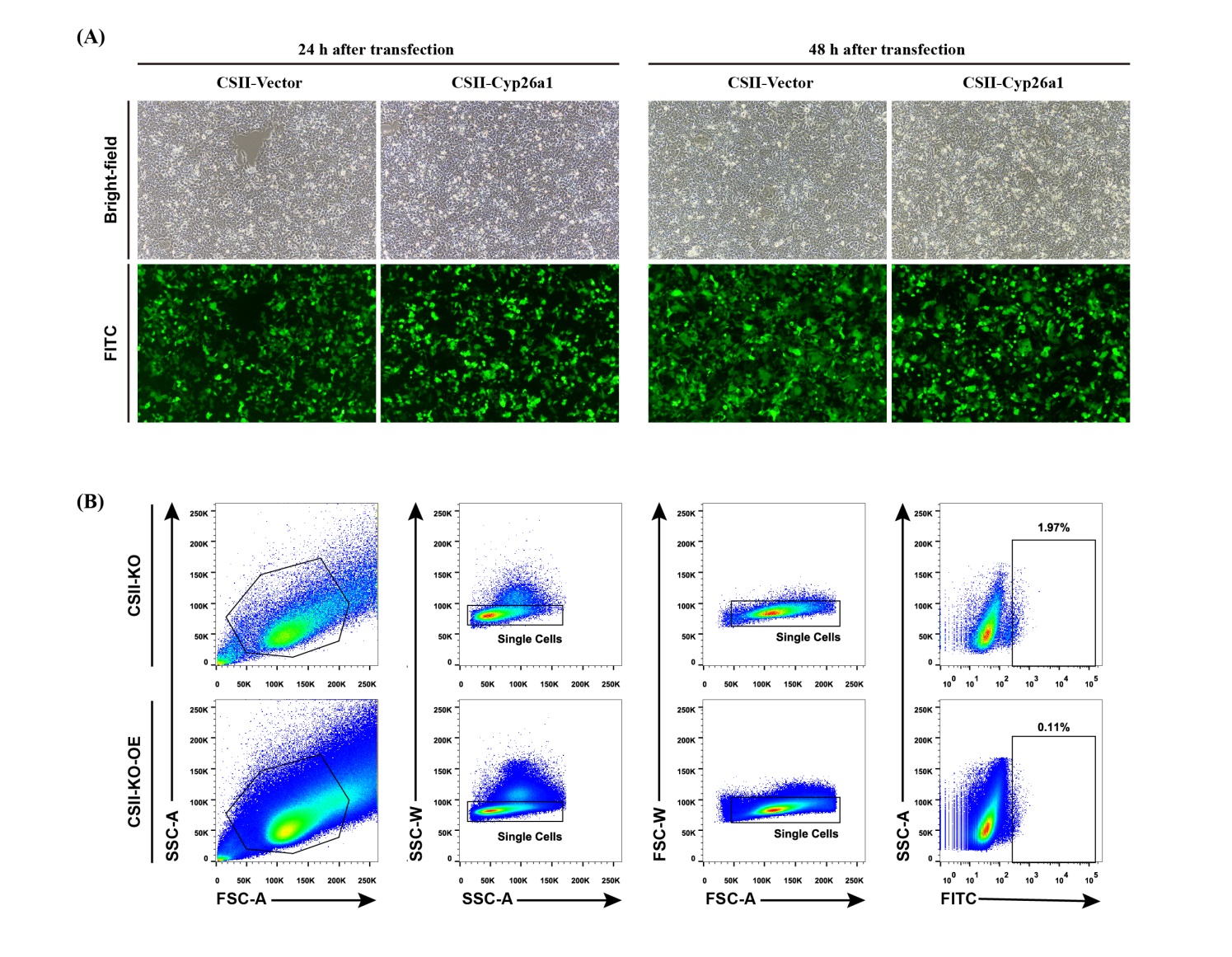


**Supplementary Figure 4** The transfection efficiency of HEK 293T cells and the percentage of YFP-positive RAW264.7 cells in Lentivirus Production and Infection. (A) Assessment of YFP-positive HEK-293T cells by fluorescence microscopy at 24 h and 48 h after transfection. **(B)** Infection efficiency analysis of YFP-positive Raw264.7 cells by FCM at 48 h after lentiviral infection.


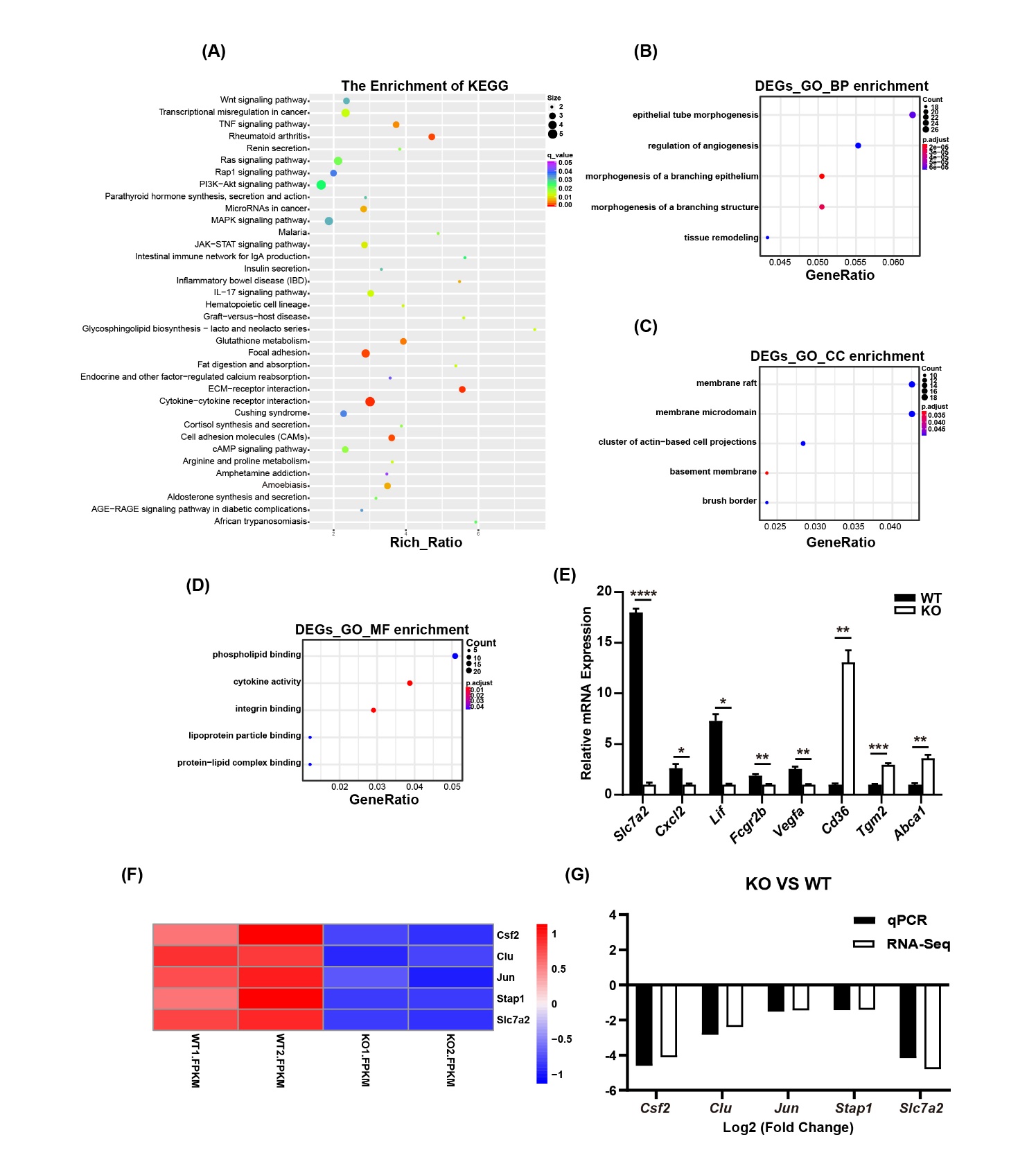


**Supplementary Figure 5** GO and KEGG analysis of DEGs in WT and KO Raw264.7 cells. **(A)** The top 36 significantly enriched KEGG pathways were listed based on the ranking of their adjusted p-values. **(B)** Top 5 enriched GO terms in the biological processes (BP). **(C)** Top 5 enriched GO terms in the molecular functions (MF). **(D)** Top 5 enriched GO terms in the cellular components (CC). **(E)** qPCR analysis of DEGs in WT and KO Raw264.7 cells (n=3). **(F)** Z-scores of genes associated with macrophages polarization in DGEs. **(G)** Comparison of validated qPCR assays with RNA-Seq for genes related to macrophage polarization in DGEs.

**Supplement Table 1 | Primers used for qPCR analysis**

| Gene name | Product length (bp) | Sequences (5' - 3') |
| --- | --- | --- |
| hGAPDH | 315bp | F: AGCCACATCGCTCAGACA  R: TGGACTCCACGACGTACT (1) |
| hCYP26A1 | 217bp | F: GCCAGTGCAGCCACATCTCT  R: GAGCAACCCGAAACCCTCCT |
| m*Gapdh* | 123bp | F: AGGTCGGTGTGAACGGATTTG  R: TGTAGACCATGTAGTTGAGGTCA (2) |
| m*Cyp26a1* | 218bp | F: CTGATTGAGCACTCGTGGGA  R: TCTAACTTGTTGTCTTGATTGCTCT |
| m*Nos2* | 117bp | F: TGTCCCAGCAATGGGCAGAC  R: GGCGCAGAACTGAGGGTACA |
| m*Il1b* | 192bp | F: AAAAAGCCTCGTGCTGTCGG  R: GTGGGTGTGCCGTCTTTCAT |
| m*Il6* | 208bp | F: TGGGACTGATGCTGGTGACA  R: GCAAGTGCATCATCGTTGTTCAT |
| m*Tnfa* | 115bp | F: GCCTCTTCTCATTCCTGCTTG  R: CTGATGAGAGGGAGGCCATT (3) |
| m*Cd86* | 100bp | F: CTTACGGAAGCACCCACGAT  R: TCTCCACGGAAACAGCATCT (4) |
| m*Cd206* | 118bp | F: TGTGGTGAGCTGAAAGGTGA  R: CAGGTGTGGGCTCAGGTAGT (4) |
| m*Arg1* | 140bp | F: GGAATCTGCATGGGCAACCTGTGT  R: AGGGTCTACGTCTCGCAAGCCA (5) |
| m*Pparg* | 120bp | F: TGTGGGGATAAAGCATCAGGC  R: CCGGCAGTTAAGATCACACCTAT (6) |
| m*Il10* | 159bp | F: AAGGCAGTGGAGCAGGTGAA  R: CCAGCAGACTCAATACACAC (7) |
| m*Dll1* | 98bp | F: GCGACTGAGGTGTAAGATGGAA  R: TCTCAGCAGCATTCATCGGG (8) |
| m*Csf2* | 211bp | F: TACAGCCTCTCAGCACCCAC  R: AATTGCCCCGTAGACCCTGC |
| m*Cd36* | 173bp | F: ACCCAGATGACGTGGCAAAG  R: TCCAACAGACAGTGAAGGCTC |
| m*Tgm2* | 137bp | F: GAAACTGGTGCTGCGTCGTG  R: CCTTGGTCCCTGCCTCTTCA |
| m*Abca1* | 195bp | F: CTGTACGCCTGAGCTACCCA  R: ACAGGCGAGACACGATGGAC |
| m*Cxcl2* | 125bp | F: GCGCTGTCAATGCCTGAAGA  R: TTTGACCGCCCTTGAGAGTG (9) |
| m*Lif* | 212bp | F: TGCCCTTACTGCTGCTGGTT  R: CACGTTGTTGGGAAACGGCT |
| m*Fcgr2b* | 170bp | F: CCAGGTCCAAGCCAGCTACA  R: GCGTGATGGTTTCCCCTTCC |
| m*Vegfa* | 198bp | F: CCCACGACAGAAGGAGAGCA  R: GCACTCCAGGGCTTCATCGT |
| m*Clu* | 220bp | F: GGCTGACAGAGCAGTACAAGGA (10) |
|  |  | R: AGCTTCACCACCACCTCAGT |
| m*Jun* | 119bp | F: TGGGCACATCACCACTACAC |
|  |  | R: TCTGGCTATGCAGTTCAGCC |
| m*Stap1* | 149bp | F: GGCGAAAAAGCCCCCGAAAC |
|  |  | R: TCCCTCTCAGCTCCGTCCA |
| m*Slc7a2* | 128bp | F: GTCATGGTGGCTGGGTTTGT |
|  |  | R: CCAGCCCCGTAGATGCTTGT |

Note: F, forward; R, reverse; h, human; m, mouse.

1. Tang F, Tang S, Guo X, Yang C and Jia K. CT45A1 siRNA silencing suppresses the proliferation, metastasis and invasion of lung cancer cells by downregulating the ERK/CREB signaling pathway. *Mol Med Rep*. (2017) 16:6708-14. doi: 10.3892/mmr.2017.7466

2. Xin Q, Li J, Dang J, Bian X, Shan S, Yuan J, et al. miR-155 Deficiency Ameliorates Autoimmune Inflammation of Systemic Lupus Erythematosus by Targeting S1pr1 in Faslpr/lpr Mice. *J Immunol*. (2015) 194:5437-45. doi: 10.4049/jimmunol.1403028

3. Yamakawa I, Kojima H, Terashima T, Katagi M, Oi J, Urabe H, et al. Inactivation of TNF-alpha ameliorates diabetic neuropathy in mice. *Am J Physiol Endocrinol Metab*. (2011) 301:E844-52. doi: 10.1152/ajpendo.00029.2011

4. Shaul ME, Bennett G, Strissel KJ, Greenberg AS and Obin MS. Dynamic, M2-like remodeling phenotypes of CD11c+ adipose tissue macrophages during high-fat diet--induced obesity in mice. *Diabetes*. (2010) 59:1171-81. doi: 10.2337/db09-1402

5. Duque-Correa MA, Kuhl AA, Rodriguez PC, Zedler U, Schommer-Leitner S, Rao M, et al. Macrophage arginase-1 controls bacterial growth and pathology in hypoxic tuberculosis granulomas. *Proc Natl Acad Sci U S A*. (2014) 111:E4024-32. doi: 10.1073/pnas.1408839111

6. Fonken LK, Workman JL, Walton JC, Weil ZM, Morris JS, Haim A, et al. Light at night increases body mass by shifting the time of food intake. *Proc Natl Acad Sci U S A*. (2010) 107:18664-9. doi: 10.1073/pnas.1008734107

7. Feng P, Chai J, Zhou M, Simon N, Huang L and Wang H. Interleukin-10 is produced by a specific subset of taste receptor cells and critical for maintaining structural integrity of mouse taste buds. *J Neurosci*. (2014) 34:2689-701. doi: 10.1523/JNEUROSCI.3074-13.2014

8. Robinson SC, Klobucar K, Pierre CC, Ansari A, Zhenilo S, Prokhortchouk E, et al. Kaiso differentially regulates components of the Notch signaling pathway in intestinal cells. *Cell Commun Signal*. (2017) 15:24. doi: 10.1186/s12964-017-0178-x

9. Inui M, Ishida Y, Kimura A, Kuninaka Y, Mukaida N and Kondo T. Protective roles of CX3CR1-mediated signals in toxin A-induced enteritis through the induction of heme oxygenase-1 expression. *J Immunol*. (2011) 186:423-31. doi: 10.4049/jimmunol.1000043

10. Xu Z, Bu Y, Chitnis N, Koumenis C, Fuchs SY and Diehl JA. miR-216b regulation of c-Jun mediates GADD153/CHOP-dependent apoptosis. *Nat Commun*. (2016) 7:11422. doi: 10.1038/ncomms11422
